# Hope on, Hope Ever

**Published:** 1885-11-20

**Authors:** 


### HOPE ON, HOPE EVER.

Some of the medical journals of this country, that have occupied a neutral ground throughout the agitation of the International Congress question for the past six months. are now taking their position in favor of the existing order of things, and we have reason to believe that measures are in progress which will restore those men who are desirable for the work to their places upon the forces of the American Medical Association, for contributing to the interest of the International meeting in Washington. The fixed purpose of those who had at heart the honor and advancement of the medical profession of the United States has been to go forward with the organization as planned by the American Medical Association, and the refusal of a portion of our prominent medical men to co-operate in the preliminary operations has not materially interfered with the general programme. Though it has necessitated various modifications in the details, the substitutes appointed for the various official positions are likely to prove as efficient in the discharge of their several duties as those originally nominated for these honorable positions. We congratulate the working corps of zealous men who have stood firmly at their posts during the trying ordeal, and feel assured that success will finally crown their efforts in behalf of the International Medical Congress.

